# Evaluating *ZNF217* mRNA Expression Levels as a Predictor of Response to Endocrine Therapy in ER+ Breast Cancer

**DOI:** 10.3389/fphar.2018.01581

**Published:** 2019-01-25

**Authors:** Julie A. Vendrell, Jérôme Solassol, Balázs Győrffy, Paul Vilquin, Marta Jarlier, Caterina F. Donini, Laurent Gamba, Thierry Maudelonde, Philippe Rouanet, Pascale A. Cohen

**Affiliations:** ^1^Univ Lyon, Université Claude Bernard Lyon 1, INSERM U1052, CNRS 5286, Centre Léon Bérard, Centre de Recherche en Cancérologie de Lyon, Lyon, France; ^2^Département de Pathologie et Oncobiologie, Laboratoire de Biologie des Tumeurs Solides, CHU Montpellier, University of Montpellier, Montpellier, France; ^3^Institut de Recherche en Cancérologie de Montpellier (IRCM), INSERM U1194, University Montpellier, Montpellier, France; ^4^2nd Department of Paediatrics, Semmelweis University, Budapest, Hungary; ^5^MTA TTK Lendület Cancer Biomarker Research Group, Institute of Enzymology, Budapest, Hungary; ^6^Biometrics Unit, Institut du Cancer de Montpellier, University of Montpellier, Montpellier, France; ^7^Département de Chirurgie Oncologique, Institut du Cancer de Montpellier, University of Montpellier, Montpellier, France

**Keywords:** breast cancer, *ZNF217*, endocrine therapy, clinical response, predictive biomarker

## Abstract

*ZNF217* is a candidate oncogene with a wide variety of deleterious functions in breast cancer. Here, we aimed at investigating in a pilot prospective study the association between *ZNF217* mRNA expression levels and the clinical response to neoadjuvant endocrine therapy (ET) in postmenopausal ER-positive (ER+) breast cancer patients. Core surgical biopsy samples before treatment initiation and post-treatment were obtained from 68 patients, and Ki-67 values measured by immunohistochemistry (IHC) were used to identify responders (*n* = 59) and non-responders (*n* = 9) after 4 months of ET. We report for the first time that high *ZNF217* mRNA expression level measured by RT-qPCR in the initial tumor samples (pre-treatment) is associated with poor response to neoadjuvant ET. Indeed, the clinical positive response rate in patients with low *ZNF217* expression levels was significantly higher than that in those with high *ZNF217* expression levels (*P* = 0.027). Additionally, a retrospective analysis evaluating *ZNF217* expression levels in primary breast tumor of ER+/HER2-/LN0 breast cancer patients treated with adjuvant ET enabled the identification of poorer responders prone to earlier relapse (*P* = 0.013), while *ZNF217* did not retain any prognostic value in the ER+/HER2-/LN0 breast cancer patients who did not receive any treatment. Altogether, these data suggest that *ZNF217* expression might be predictive of clinical response to ET.

## Introduction

In recent years, studies investigating neoadjuvant therapies have emerged improving both patient management by providing a means of performing less extensive surgery and our understanding of tumor biology and response to treatment (for review, Charehbili et al., 2014). Neoadjuvant ET is administered to HR-positive postmenopausal patients, as recommended by the 15th St. Gallen International Breast Cancer Conference (Morigi, 2017). The main advantage of such a preoperative systemic ET is the prospect of downsizing and down-staging large tumors, thus facilitating breast-conserving surgical interventions. Despite the use of standard biomarkers, the heterogeneity of response to therapy still represents a challenge to clinicians in terms of selecting the most suitable neoadjuvant therapy. Thus, there is an urgent need to discover predictive biomarkers capable to identify patients who will respond to neoadjuvant ET.

We previously described that high expression levels of *ZNF217*, a candidate oncogene, are associated with poor prognosis, shorter RFS in breast cancer (Vendrell et al., 2012; Bellanger et al., 2017). A functional crosstalk exists between *ZNF217* and ER signaling (Nguyen et al., 2014), representing a potential mechanism to escape ET. Most interestingly, high *ZNF217* expression levels confer resistance to ET in ER+ breast cancer cell lines, and *ZNF217* expression silencing is associated with reversion of such resistance (Nguyen et al., 2014). Furthermore, a decrease in Ki-67 levels during neoadjuvant ET (considered alone or as part of a Preoperative Endocrine Prognostic Index) was shown to predict response to ET (Dowsett et al., 2005, 2007; Ellis et al., 2011, 2017; Iwamoto et al., 2017). The aim of this pilot study is to investigate the predictive value of *ZNF217* mRNA levels for response to neoadjuvant ET in patients with ER+ breast cancer.

## Materials and Methods

### Study Design

This was a prospective neoadjuvant ET study on breast cancers expressing the estrogen receptor (ER+) and having a clinical size exceeding 2 cm (T2). This study has been approved by the local ethics committee (Institut du Cancer de Montpellier, France). Patients were informed that their data could be used for research; all the patients signed an informed consent form and the study was conducted in accordance with the Declaration of Helsinki principles. A total of 111 patients were treated for 4 months with neoadjuvant ET (letrozole 2.5 mg/day or tamoxifen 20 mg/day), before being subjected to resection surgery (see Supplementary Material). The response to treatment was evaluated by monitoring the evolution of a biological marker of proliferation (Ki-67) before (initial tumor) and after 4 months of ET. Investigation of *ZNF217* mRNA expression levels was also conducted in the initial breast tumor and in the post-treatment tumor samples.

### Sample Collection

Three micro-biopsies were collected per patient: one for histopathological diagnosis and the other two were frozen in liquid nitrogen until further use. These tissues were later used for RNA extraction and *ZNF217* mRNA expression analysis, respecting post-therapeutic medical diagnostic requirements. Moreover, IHC examination was carried out to assess the statuses of ER, PR, HER2, and Ki-67. Ki-67 IHC values were measured pre- and post-treatment for each patient and used to discriminate between responders and non-responders (Dowsett et al., 2007). Patients displaying a ΔKi-67 (Ki-67 IHC value post-treatment – Ki-67 IHC value pre-treatment) ≤0 were designated to be responders, while patients with ΔKi-67 >0 were non-responders.

### RNA Extraction and Real-Time Quantitative PCR (RT-qPCR)

Total RNA was extracted from frozen biopsies using the RNeasy Mini Kit (Qiagen, Hilden, Germany). After checking RNA quality, 68 tumor samples were deemed suitable for expression analysis (59 responders and nine non-responders) (Supplementary Table 1). Reverse-transcription and RT-qPCR measurements were performed as described in the Supplementary Material. A *P*-value of ≤0.05 was considered to be statistically significant (Statgraphics^TM^ Software). ROC-AUC was investigated using the SPSS^TM^ Software.

### The Kaplan-Meier Plotter (KMP) Breast Cancer Cohort

The KMP cohort investigation resulted from a meta-analysis of gene-expression profiles from 2,978 primary breast cancer specimens who had not received any therapy before surgery and with known adjuvant therapy and clinical follow-up (Gyorffy and Schafer, 2009). The SPSS^TM^ Software was used to assess the prognostic value of *ZNF217* or *Ki-67* mRNA expression (univariate analysis). Data were divided into two groups with either high or low expression values according to the median value. Candidate prognostic factors for RFS with a 0.1 significance level in univariate analysis were entered in a multivariate Cox model, and a backward selection procedure was used to determine independent prognostic markers.

## Results

*ZNF217* mRNA expression levels were not correlated with Ki-67 values, neither in the initial breast tumor (pre-treatment) (*r* = -0.169, *P* = 0.17), nor in the post-treatment samples (*r* = -0.026, *P* = 0.83), nor with the ΔKi-67 values (*r* = -0.136, *P* = 0.26), thus ruling out that investigating *ZNF217* expression levels was merely a surrogate markers of Ki-67 expression (Spearman test).

**FIGURE 1:**
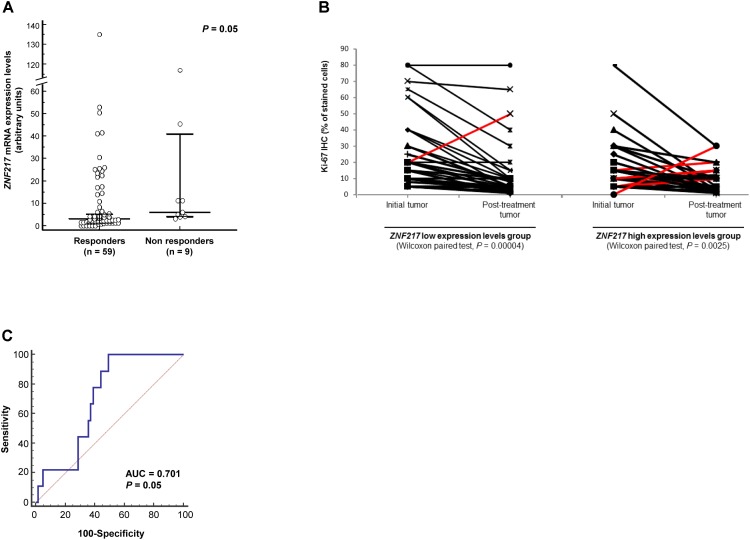
High *ZNF217* mRNA expression levels are associated with poor neoadjuvant ET response. **(A)** Dot plot representing *ZNF217* mRNA expression levels in the initial breast tumors of responders or non-responders. Medians and 95% confidence intervals are shown for each group. **(B)** Changes in the Ki-67 expression for patients in the initial tumor and post-treatment tumor according to the mRNA expression levels of *ZNF217.* Red lines correspond to the non-responders (displaying increased ΔKi-67). **(C)** Receiver operating characteristic (ROC) curve for *ZNF217* mRNA expression levels. AUC, area under curve.

In responders (*n* = 59) and in non-responders (*n* = 9), *ZNF217* expression was associated with response to neoadjuvant ET, since *ZNF217* mRNA expression levels tended to be significantly higher (*P* = 0.05) in the initial breast tumor in patients who did not respond to neoadjuvant ET (median = 5.98) than those who did (median = 3.01) (Figure 1A).

Fisher’s exact test was used to investigate the association between the dichotomized clinical response measures and the *ZNF217* molecular marker. The 68 patients were separated in two groups, based on the median *ZNF217* expression value. The positive clinical response rate in the low *ZNF217* expression level group was significantly higher (*P* = 0.027) than that in the high *ZNF217* expression level group, with high *ZNF217* expression levels being associated with the absence of response to neoadjuvant ET (Supplementary Table 2).

The *ZNF217* low and high expression level groups were comparable in terms of Ki-67 values in the initial tumor (pre-treatment) (*P =* 0.20, data not shown). Changes in Ki-67 for individual patients before and after neoadjuvant ET response are shown in Figure 1B. In the *ZNF217* low expression levels group (Wilcoxon signed-rank test, *P* < 0.00004), only one patient was ET-resistant (displaying increased Ki-67 level), while 33/34 patients were responders (Figure 1B). In the *ZNF217* high expression level group (Wilcoxon signed-rank test, *P* < 0.0025), eight patients displayed an increase in Ki-67 (ET-resistant patients), whereas 26 patients were responders (Figure 1B).

Although our study is exploratory with a limited sample size (*n* = 68) and with limited relapse events (*n* = 9), we assessed the predictive power of *ZNF217* expression level for response to ET. The AUC was 0.701 (95% confidence interval: 0.563–0.838, *P* = 0.05), which represents a good/moderate discriminatory accuracy for a model including few events (*n* = 9) (Figure 1C). Based on the ROC curve, the discriminating sensitivity and specificity were 100 and 51%, respectively.

A number of studies have suggested that post-treatment biomarkers (such as Ki-67 and ER) could have a better prognostic value than pre-treatment biomarkers, and investigating biomarkers in post-treatment samples is thus of interest (Dowsett et al., 2007; Ellis et al., 2008; Chia et al., 2010). In post-treatment tumor samples, no differences in *ZNF217* expression levels were observed between responders and non-responders (*P* = 0.4, Mann-Whitney test, data not shown), suggesting that assessing *ZNF217* expression levels in the initial tumor before ET is the most informative.

**FIGURE 2:**
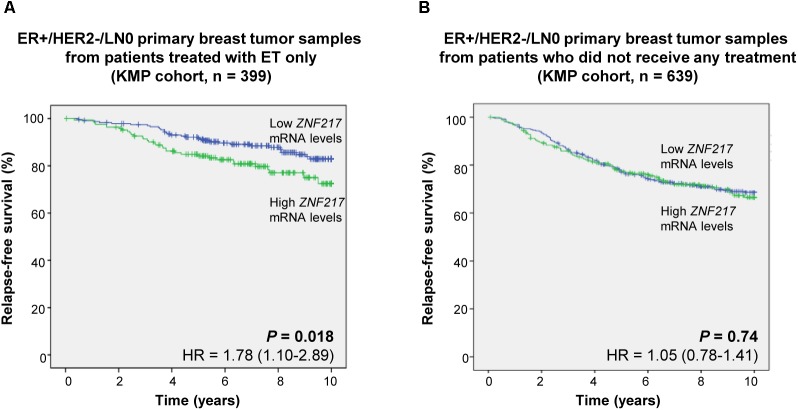
High *ZNF217* mRNA expression levels are associated with earlier relapse for patients treated with ET only. Kaplan-Meier analyses for relapse-free survival (RFS) are shown for ER+/HER2–/LN0 breast cancer patients **(A)** treated with adjuvant ET only and **(B)** who did not receive any treatment.

To support our finding, we then hypothesized that if *ZNF217* expression retains any predictive value for ET response in the neoadjuvant setting, then, the biomarker value of *ZNF217* would be different between ER+ breast cancer patients treated with adjuvant ET only and patients who did not received any treatment. We thus performed a retrospective analysis of gene-expression array data from 2,978 breast cancer patients (KMP cohort). In this cohort, we have previously demonstrated that high levels of *ZNF217* mRNA expression levels were strongly and significantly associated with shorter RFS (*P* < 10^-9^, Nguyen et al., 2014). Strikingly, when considering the ER+/HER2-/LN0 patients, high *ZNF217* mRNA levels were predictive of earlier relapse for patients treated with adjuvant ET only (*n* = 399, *P* = 0.018, univariate analysis), but not for non-treated patients (*n* = 639, *P* = 0.74, univariate analysis) (Figure 2). In ER+/HER2–/LN0 patients, *ZNF217*, and *Ki-67* mRNA expression levels were not correlated (*r* = -0.07, *P* = 0.14, Spearman test). Since *Ki-67* mRNA levels were almost significantly correlated with RFS in this cohort (*P* = 0.094, univariate analysis), the two factors were entered in a multivariate Cox model, and both persisted in the model showing that they are independent biomarkers (*P* < 0.1). A signature associating *ZNF217* and *Ki-67* mRNA levels displayed a prognostic value with regards to RFS (*P* = 0.01, univariate analysis). Interestingly, this signature was identified as the best fit for predicting clinical outcome of ET-treated patients (likelihood = 741.46), compared to the models integrating *ZNF217* mRNA levels (likelihood = 746.65, *P* = 0.023) or *Ki-67* mRNA levels (likelihood = 749.24, *P* = 0.005) only. Our data support that *ZNF217* is a predictive biomarker for response to ET, and that, in the ER+/HER2-/LN0 cohort, the signature including both *ZNF217*/*Ki-67* mRNA levels had the best predictive value.

## Discussion

Short-term pre-operative trials with specific groups of patients have proven to be highly promising in identifying biomarkers predictive for the efficacy of targeted anti-cancer therapies (Marous et al., 2015). Early evidence of endocrine drug effectiveness can be obtained in the pre-operative (neoadjuvant) setting by profiling baseline and on-treatment biopsy samples using the window-of-opportunity. This predictive evidence acquired during short-term neoadjuvant therapy can help in identifying individual patients who will potentially benefit from long-term adjuvant treatment enabling personalized approaches. Short-term reduction in Ki-67 is predictive of clinical response to ET (Dowsett et al., 2005, 2007; Ellis et al., 2011, 2017; Iwamoto et al., 2017). However, controversy remains regarding the reproducibility of Ki-67 measurements and international efforts are ongoing to standardize and validate Ki-67 by IHC (Polley et al., 2015; Rimm et al., 2018). An additional obstacle derives from intra-tumor heterogeneity of Ki-67 (Focke et al., 2016). Altogether, there is an urgent need for further biomarkers that might increase the accuracy of prediction of response to ET.

In a previous study, proliferation-associated genes, including cyclins, mini chromosome maintenance genes and mitotic spindle-associated genes were shown to be predictive of response to ET after 2 weeks but not before treatment (Turnbull et al., 2015). A four-gene signature measuring two genes pre-treatment and two genes after 2 weeks of treatment was shown to predict response to neoadjuvant ET in ER+ patients (Turnbull et al., 2015). The genes that predicted response to ET included two pre-treatment genes associated with immune response and apoptosis and two genes measured after 2 weeks of treatment, which were associated with proliferation. Altogether, these data suggest that transcriptomic changes that develop during treatment are representative of the drug’s mechanism of action, suggesting that suppression of proliferation is the main driver of response.

In the present pilot study, the clinical sample size used is small and included only nine non-responders. Nevertheless, we found that *ZNF217* expression levels are predictive of neoadjuvant ET response in ER+ breast cancer. *ZNF217* expression levels are not associated with Ki-67 values, neither in the initial nor in the treated tumor, ruling out that *ZNF217* could only be a surrogate marker of cell proliferation. Of utmost interest is that the predictive value of *ZNF217* expression levels seems to reside in the initial tumor and is not the reflection of transcriptional changes following ET in the treated tumors. This is the first pilot prospective study conducted in the neoadjuvant setting to relate *ZNF217* expression levels with treatment efficacy, thus suggesting that aside from its prognostic value in luminal breast cancers (Vendrell et al., 2012; Nguyen et al., 2014), *ZNF217* expression may also be predictive of response to ET. In this exploratory study, it is difficult to estimate the accuracy of *ZNF217* mRNA levels for predicting response to ET, due to the low numbers of non-responders (9 out of 68). However, while obtained in a small cohort, our preliminary data are encouraging and need to be extended to a larger cohort for validation.

Interestingly, evaluating *ZNF217* expression levels in the primary breast tumor of ER+/HER2-/LN0 breast cancer patients treated by adjuvant ET led to the identification of poorer responders prone to earlier relapse, while in ER+/HER2-/LN0 breast cancer patients who did not receive any treatment the association between *ZNF217* expression and RFS was not significant. Previous studies reported multi-gene genomic assays predicting response to neoadjuvant ET (Turnbull et al., 2015; Iwata et al., 2018), and we speculate that these coupling with the *ZNF217* biomarker might improve their predictive performance. Indeed, we herein demonstrated in the ER+/HER2–/LN0 cohort that combining *ZNF217* and *Ki-67* expression levels was more performant at predicting relapse under ET, than each of these biomarkers taken individually.

Altogether, these data support the idea that in the luminal breast cancer subclass, *ZNF217* expression levels relate to ET response and provide a novel candidate biomarker. Finally, there are several ongoing trials investigating the combination of ET and other targeted therapies to prevent/reverse endocrine resistance. The PI3K/mTOR pathway, CDK4/6, HDAC, and immune checkpoints are the most promising and widely investigated targets (Rugo et al., 2016). Further studies are needed to delineate whether the ER+/*ZNF217*_high_ breast cancer subpopulation might benefit from combining ET with another therapy.

## Author Contributions

PR and TM designed and supervised the neoadjuvant clinical trial. JV, PV, and LG performed the experiments. JV, PV, and CD performed the RT-qPCR data analysis. MJ performed the clinical data analysis. JV and BG performed the retrospective *in silico* analysis. PR, TM, and PC conceived and supervised the study. JV, JS, TM, and PC wrote the manuscript.

## Conflict of Interest Statement

The authors declare that the research was conducted in the absence of any commercial or financial relationships that could be construed as a potential conflict of interest.

## ABBREVIATIONS

ER+: ERα-positive
ERα: estrogen receptor alpha
ET: endocrine therapy
HR: hormone receptor
IHC: immunohistochemistry
LN0: no invaded lymph nodes
PR: progesterone receptor
RFS: relapse-free survival
RT-qPCR: real-time quantitative PCR
