# Supplementary material for: Evaluating ZNF217 mRNA Expression Levels as a Predictor of Response to Endocrine Therapy in ER+ Breast Cancer
Source: Front Pharmacol. 2019 Jan 25;9:1581. doi: 10.3389/fphar.2018.01581 (PMC6355665; doi:10.3389/fphar.2018.01581)
Supplement: Supplementary file 1 [file Data_Sheet_1.docx]

**SUPLEMENTARY MATERIAL**

**Patients**

Postmenopausal women over the age of 65 with previously untreated ER+ breast adenocarcinoma and with a primary tumor of 2 cm or greater (T2) were eligible for our study. ER positivity was determined on pre-treatment biopsies by radio-ligand assay (> 10 fm/ml) or by immunohistochemistry (IHC) (> 10% of stained cells). Women who were not eligible for the trial included those (i) with ER– breast cancers at diagnosis, (ii) with tumors < T2 or less than 1 cm diameter after ultrasound, (iii) who had previously had a breast cancer or having metastatic cancer or inflammatory breast cancer, (iv) on hormone replacement therapy (HRT off delay: 15 days) for their menopause or any other (equivalent to HRT) treatment capable of modifying the expression of genes regulated by estrogen, (vi) with a contraindication for treatment with tamoxifen or letrozole, and finally, (vii) with clinically metastatic lymphadenopathy and eligible for chemotherapy.

**Immunohistochemistry (IHC)**

Immunostaining was performed centrally with a Ventana NeXes automat (Ventana Medical Systems Inc, Tucson, AZ). Tumors were stained for ER (Clone 6F11, Novocastra; dilution 1:40), PR (Clone 1A6, Novocastra, Newcastle Upon Tyne, United Kingdom; dilution 1:30), Ki-67 (Clone MIB1, Dako, Glostrup, Denmark; dilution 1:100), and HER2 (Ab A0485, Dako; dilution 1:250), according to manufacturer’s recommendations. The visualization was performed with an Envision Flex kit (Dako).

**Real-time quantitative PCR (RT-qPCR)**

Total RNA (500 ng) were reversed-transcribed using oligodT primers, and RT-qPCR measurements were performed in triplicate using a LightCycler 480 (Roche, Meylan, France) in combination with the LightCycler 480 DNA SYBR Green I Master (Roche). *ZNF217* gene expression measurements were performed as previously described (Vendrell et al. 2012) and were normalized against the expression of the *RPL13A* housekeeping gene.

**Supplementary Table 1**: Baseline characteristics of responders and non-responders

| **Baseline data** | | **Responders** | | **Non-Responders** | | **Total** | |  |
| --- | --- | --- | --- | --- | --- | --- | --- | --- |
|  |  | **N** | **%** | **N** | **%** | **N** | **%** | **p^(2)^** |
| Age (years) |  |  |  |  |  |  |  | 0.280 |
|  | <70 | 21 | 35.59 | 1 | 11.11 | 22 | 32.35 |  |
|  | ]70-80] | 24 | 40.68 | 6 | 66.67 | 30 | 44.12 |  |
|  | ]80-90] | 14 | 23.73 | 2 | 22.22 | 16 | 23.53 |  |
| BMI (kg/m2) |  |  |  |  |  |  |  | 0.401 |
|  | Underweight | 1 | 1.69 | 0 |  | 1 | 1.47 |  |
|  | Normal weight | 21 | 35.59 | 3 | 33.33 | 24 | 35.29 |  |
|  | Overweight | 24 | 40.68 | 2 | 22.22 | 26 | 38.24 |  |
|  | Obesity | 13 | 22.03 | 4 | 44.44 | 17 | 25.00 |  |
| erB2 |  |  |  |  |  |  |  | 0.738 |
|  | 0 | 18 | 30.51 | 3 | 33.33 | 21 | 30.88 |  |
|  | + | 33 | 55.93 | 5 | 55.56 | 38 | 55.88 |  |
|  | ++ | 5 | 8.47 | 0 |  | 5 | 7.35 |  |
|  | +++ | 3 | 5.08 | 1 | 11.11 | 4 | 5.88 |  |
| Tumor diameter (cm) ^(1)^ | |  |  |  |  |  |  | 0.936 |
|  | >0.5 et <=1 | 5 | 8.47 | 0 |  | 5 | 7.35 |  |
|  | >1 et <=2 | 34 | 57.63 | 5 | 55.56 | 39 | 57.35 |  |
|  | >2 et <=3 | 16 | 27.12 | 3 | 33.33 | 19 | 27.94 |  |
|  | >3 et <=5 | 4 | 6.78 | 1 | 11.11 | 5 | 7.35 |  |
| Grade SBR |  |  |  |  |  |  |  | 0.100 |
|  | I | 9 | 15.52 | 0 |  | 9 | 13.43 |  |
|  | II | 36 | 62.07 | 9 | 100.00 | 45 | 67.16 |  |
|  | III | 13 | 22.41 | 0 |  | 13 | 19.40 |  |
|  | Missing | 1 |  | 0 |  | 1 |  |  |

^(1)^Maximum tumor diameter assessed by echography

^(2)^Fisher’s exact test

**Supplementary Table 2:** Proportion of patients that responded or not to neoadjuvant ET in the *ZNF217* low expression level group and the *ZNF217* high expression level group.

|  | *ZNF217* low mRNA levels group (N = 34) | |  | *ZNF217* high mRNA levels group (N = 34) | |  | *P^(1)^* |
| --- | --- | --- | --- | --- | --- | --- | --- |
|  | *N* | % |  | *N* | % |  |  |
| Responders | 33 | 97.0 |  | 26 | 76.5 |  | 0.027 |
| Non-responders | 1 | 3.0 |  | 8 | 23.5 |  |  |

^(1)^Fisher’s exact test
